# The potential role of ICU capacity strain in COVID-19 mortality: comparison between first and second waves in Pavia, Italy

**DOI:** 10.1186/s44158-021-00007-6

**Published:** 2021-10-22

**Authors:** Francesco Mojoli, Sara Cutti, Silvia Mongodi, Raffaele Bruno, Antonio Di Sabatino, Angelo Guido Corsico, Carlo Marena

**Affiliations:** 1 Department of Clinical-surgical, Diagnostic and Pediatric Sciences, Unit of Anesthesia and Intensive Care, University of Pavia, Pavia, Italy; 2 Anesthesia and Intensive Care, San Matteo Hospital, Pavia, Italy; 3 Unit of Direzione Medica di Presidio, S. Matteo Hospital, Pavia, Italy; 4 Rianimazione I, Fondazione IRCCS Policlinico S. Matteo, DEA piano -1, Viale Golgi 19, 27100 Pavia, Italy; 5 Department of Clinical-surgical, Diagnostic and Pediatric Sciences, University of Pavia, Pavia, Italy; 6 Division of Infectious Disease, San Matteo Hospital, Pavia, Italy; 7 Department of Internal Medicine, San Matteo Hospital, University of Pavia, Pavia, Italy; 8 Center for Diagnosis of Inherited α1-Antitrypsin Deficiency, Department of Internal Medicine and Therapeutics, Pneumology Unit, San Matteo Hospital, University of Pavia, Pavia, Italy

**Keywords:** COVID-19 waves, ICU organization, ICU capacity, ICU preparedness, COVID-19 mortality

To the Editor,

In novel coronavirus disease (COVID-19) pandemic, a high mortality rate was reported during wave 1, particularly when COVID-19 load exceeded 100% ICU capacity [1]. With better knowledge of the disease [2, 3], a lower mortality was expected in wave 2; however, similar mortalities were reported for hospital/ICU populations [4]. Within the same wave, in-hospital mortality was lower once past the peak of hospital affluence [1, 5], suggesting a role of ICU facilities’ availability. However, their actual role remains unclear since mortality was high also in non-overwhelmed healthcare systems [6].

To test if disproportion between ICU facilities and hospitalized patients impact COVID-19 mortality, we compared the first 8 weeks of waves 1 vs. 2 in Pavia (Lombardy, Italy). ICU-timing (time from hospital to ICU admission), percentage of COVID-19 hospitalized patients admitted to ICU, and percentage of intubated patients in ICU were considered ICU capacity strain’s markers. All patients during wave 2 received steroids as appropriate [3]. Local ethic committee approved the study.

Patients’ characteristics are in Table 1. In wave 1, a steep increase of ICU COVID-19 patients reached a peak of 64 on day 34 (Fig. 1A); a plateau phase lasted 14/56 days (25.0%); thereafter, a reduction was observed. In wave 2 (Fig. 1B), a slower increase achieved a lower peak (54 patients) on day 40 and lasted 4/56 days (7.1%; *p*=0.010).

**Table 1:** Features of the patients admitted to general ward and to ICU during the first and the second COVID-19 waves in Pavia

		1st + 2nd waves (***N*** = 1736)	1st wave (***N*** = 1062)	2nd wave (***N*** = 674)	***P*** value
**Ward patients**	*n* (%)	1478 (85.1)	923 (86.9)	555 (82.3)	**0.0104**
Male	*n* (%)	914 (61.8)	555 (60.1)	359 (64.7)	0.0866
Age	Years	69.6 ± 15.2	69.8 ± 15.4	69.3 ± 15.0	0.5154
Hospital stay	Days	11.9 ± 10.8	11.3 ± 10.6	13.0 ± 11.1	**0.0027**
Hospital deaths	*n* (%)	416 (28.1)	307 (33.3)	109 (19.6)	**<0.0001**
**ICU patients**	*n* (%)	258 (14.9)	139 (13.1)	119 (17.7)	**0.0104**
Male	*n* (%)	215 (83.3)	117 (84.2)	98 (82.4)	0.7392
Age	Years	61.9 ± 11.2	61.4 ± 11.1	62.5 ± 11.5	0.4323
Invasive mechanical ventilation	*n* (%)	224 (86.8)	134 (96.4)	90 (75.6)	**<0.0001**
ICU timing	Hours	74.8 ± 92.5	89.8 ± 90.6	57.3 ± 92.0	**0.0047**
ICU stay	Days	26.8 ± 23.2	26.6 ± 23.9	27.1 ± 22.4	0.8685
ICU deaths	*n* (%)	102 (39.5)	68 (48.9)	34 (28.6)	**0.0009**
Hospital stay	Days	37.3 ± 26.3	36.3 ± 28.4	38.5 ± 23.5	0.5089
Hospital deaths	*n* (%)	104 (40.3)	68 (48.9)	36 (30.3)	**0.0033**

*ICU* intensive care unit*.* Data are expressed as *n* (%) or mean ± standard deviation. In bold: significant *p* values <0.05

**Fig. 1:**
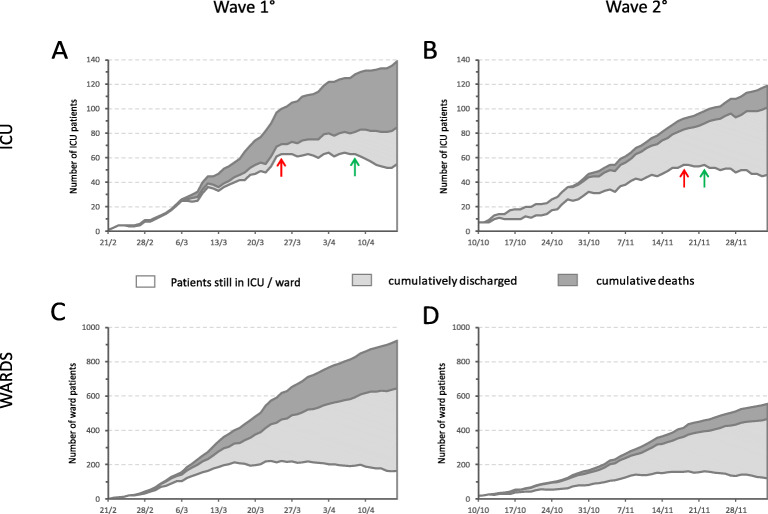
The first 8 weeks of the two pandemic waves in ICU and in the wards: this timeframe was representative of the critical phase for our healthcare system, including rapid increase of ICU patients up to a peak (red arrows), plateau phase, and initial decline (green arrows). **A** Wave 1 in ICU. A steep increase of ICU COVID-19 patients was observed until a peak of 64; pre-pandemic capacity was 32 beds. The peak was reached on day 34; a plateau phase persisted until day 48; thereafter, a reduction was observed. After 8 weeks, 139 patients had been admitted to ICU (13.1% of hospital admissions) with 55 (39.6%) patients still in ICU, 30 (21.6%) discharged and 54 (38.8%) deceased. At this time, mortality was 54/84 (64.3%) in ICU patients. **B** Wave 2 in ICU. The initial increase was slower, and a lower peak (54 ICU patients) was achieved on day 40; a plateau phase lasted until day 44, when the decline started. After 8 weeks, 119 patients had been admitted to ICU (17.7% of hospital admissions, *p*=0.0104 vs. wave 1) with 45 (37.8%) patients still in ICU, 56 (47.1%) discharged, and 18 (15.1%) cumulative deaths (*p*<0.0001 vs. wave 1). At this time, mortality was 18/74 (24.3%) in ICU patients (*p*<0.0001 vs. wave 1). **C** Wave 1 in the wards. After 8 weeks, 923 patients had been admitted (86.9% of hospital admissions) with 175 (19.0%) patients still in the ward, 475 (51.5%) discharged, and 273 (29.6%) deceased. At this time, mortality was 273/748 (36.5%) in ward patients. **D** Wave 2 in the wards. After 8 weeks, 555 patients had been admitted (82.3% of hospital admissions, p=0.0104 vs. wave 1) with 134 (24.1%) patients still in the ward, 334 (60.2%) discharged and 87 (15.7%) deceased (p<0.0001 vs. wave 1). At this time, mortality was 87/421 (20.7%) in ward patients (p<0.0001 vs. wave 1)

At day 56 of wave 1, patients admitted to ICU were 139, ICU mortality was 54/84 (64.3%), patients still in ICU were 55 (39.6%), and their follow-up ICU mortality was 14/55 (25.5%), lower than in the beginning of the same wave (*p*<0.0001).

At day 56 of wave 2, patients admitted to ICU were 119, ICU mortality was 18/74 (24.3%; *p*<0.0001 vs. wave 1), patients still in ICU were 45 (37.8%), and their follow-up ICU mortality was 16/45 (35.6%), similar to the first 8 weeks (*p*=0.2133). Findings in ward patients are displayed in Fig. 1C, D.

In waves 1 and 2, hospital mortality was in overall ICU patients 48.9% and 30.3% (*p*=0.0033), in intubated patients 50.7 and 36.7% (*p*=0.0410), in ward patients 33.3% and 19.6% (*p*<0.0001), respectively.

Wave 2 determined a lower ICU strain: patients that could be treated in ICU were 17.7 vs. 13.1% (relative increase 35.1%; *p*=0.0104); ICU-timing was shorter (57±92 vs. 90±91 h; p=0.0047), with patients admitted to ICU within 48 h 58.0 vs. 40.3% (*p*=0.0059); and intubation was less frequent (75.6 vs. 96.4%; *p*<0.0001).

ICU-timing was resulted in an independent risk factor for hospital mortality when adjusted for age, gender, and need of invasive ventilation (*p*<0.0001).

The improvement of ICU and ward patients’ outcome exceeded what expected from steroids’ introduction [3], supporting that other factor may have a role [5]. ICU strain was significantly higher during wave 1. Moreover, patients were admitted to ICU later, when intubation was almost unavoidable, which may increase mortality [5]. ICU-timing was an independent predictor of mortality, suggesting intensive care should be considered a time-dependent treatment for COVID-19 patients.

In conclusion, COVID-19 mortality notably decreased in wave 2 at our institution; beyond the benefits of a deeper knowledge of the disease, lower ICU capacity strain and timelier ICU admission may have played a role.

## Data Availability

The datasets used and/or analysed during the current study are available from the corresponding author on reasonable request.

## Abbreviations

COVID-19: Novel coronavirus disease
ICU: Intensive care unit
